# Supplementary material for: Behavioural effects of methylphenidate in the spontaneously hypertensive rat model of attention-deficit/hyperactivity disorder: a systematic review and meta-analysis protocol
Source: BMJ Open Sci. 2018 Oct 19;2(1):e000001. doi: 10.1136/bmjos-2018-000001 (PMC8715948; doi:10.1136/bmjos-2018-000001)
Supplement: Author's manuscript [file bmjos-2018-000001.draft_revisions.pdf]

BMJ Open Science

BMJ Open Science is committed to open peer review. As part of this commitment we make the peer review history of every article we publish publicly available.

When an article is published we post the peer reviewers' comments and the authors' responses online. We also post the versions of the paper that were used during peer review. These are the versions that the peer review comments apply to.

The versions of the paper that follow are the versions that were submitted during the peer review process. They are not the versions of record or the final published versions. They should not be cited or distributed as the published version of this manuscript.

BMJ Open Science is an open access journal and the full, final, typeset and author-corrected version of record of the manuscript is available on our site with no access controls, subscription charges or pay-per-view fees (http://openscience.bmj.com).

If you have any questions on BMJ Open's open peer review process please email info.bmjos@bmj.com

Behavioral effects of methylphenidate in an animal model of attention-deficit/hyperactivity disorder, the spontaneously hypertensive rats: a systematic review and meta-analysis protocol

Douglas Teixeira Leffa[1,2], Alana Castro Panzenhagen[3,4], Diego Luiz Rovaris[3,4,5], Iraci L.S. Torres[1,2], Luis Augusto Rohde[4,5,6], Eugenio Horacio Grevet[4,5], Gabriel Natan Pires[7]

[1]Post-Graduate Program in Medicine: Medical Sciences, School of Medicine, Universidade Federal do Rio Grande do Sul, Porto Alegre, Brazil

[2]Laboratory of Pain Pharmacology and Neuromodulation: Pre clinical studies - Pharmacology Department, Institute of Basic Health Sciences, Universidade Federal do Rio Grande do Sul, Porto Alegre, Brazil

[3]Department of Genetics, Universidade Federal do Rio Grande do Sul, Porto Alegre, Brazil.

[4]ADHD Outpatient Program, Hospital de Clínicas de Porto Alegre, Brazil

[5]Department of Psychiatry, Universidade Federal do Rio Grande do Sul, Porto Alegre, Brazil

[6]National Institute of Developmental Psychiatry for Children and Adolescents, Brazil

[7]Departamento de Psicobiologia, Universidade Federal de São Paulo – São Paulo, Brazil

Corresponding author:

Douglas Teixeira Leffa

Post-Graduate Program in Medicine: Medical Sciences - School of Medicine, UFRGS

Rua Ramiro Barcelos – 2400

90035-003 – Porto Alegre, RS, Brazil

Phone: (55-51)3308.5232

E-mail: douglasleffa@hotmail.com

Abstract

Attention-deficit/hyperactivity disorder (ADHD) is a prevalent condition related to several negative outcomes. Although a strong neurobiological basis has been demonstrated, its pathophysiology is still poorly understood. In this sense, animal studies are essential in order to unravel the neurobiological factors associated with neuropsychiatric disorders, and the absence of proper animal models may be contributing to the currently challenges in the development of new treatments. The spontaneously hypertensive rats (SHR) are the most commonly used animal model of ADHD. However, its validity, and especially its predictive validity, has been questioned. In this study we aim at summarizing the evidence for the predictive validity of the SHR as an animal model of ADHD. The current protocol discloses the background, aims and methods of a systematic review and meta-analysis of studies reporting behavioral effects of methylphenidate (MPH), the most commonly prescribed treatment for ADHD, in the SHR. In addition, we plan to evaluate how pre-defined covariates influence the effects of MPH using meta-regression and sensitivity analyses.

Keywords: ADHD, SHR, meta-analysis, methylphenidate

1. Introduction

Attention-deficit/hyperactivity disorder (ADHD) is a prevalent neurodevelopmental disorder characterized by impairing levels of hyperactivity, impulsivity and inattention (1). Observational studies have shown that ADHD is related to several negative outcomes, including, among others, decreased quality of life (2), increased number of suicide attempts (3), less socioeconomic status (4), and increased mortality due to accidents (5).

A strong neurobiological basis has been repeatedly demonstrated in ADHD. Twin studies showed that ADHD has a heritability of about 70-80% (6), and candidate gene studies suggest mainly the influence of genes related to monoaminergic neurotransmission (7). A recent mega-analysis performed with more than 1500 ADHD patients found a reduced volume in the nucleus accumbens, amygdala, caudate, hippocampus and putamen in patients with the disorder (8). In addition, a meta-analysis of fMRI studies demonstrated reduced activation in distinct cortical region during attention and impulsivity tests (9). Although ADHD neurobiological basis has been confirmed through molecular genetics and neuroimaging studies, its pathophysiology is still poorly understood.

Animal models are considered a fundamental tool to unravel the neurobiological factors associated with neuropsychiatric disorders (10). More important, the absence of proper animal models has been proposed as a main factor driving the slow advancement of new treatments for neuropsychiatric disorders (10). An animal model should present predictive, face and construct validity in order to be considered a proper model of a disease (11). In ADHD, the spontaneously hypertensive rats (SHR) are widely considered the most appropriate model (12, 13). However, there is no consensus on the validity of the SHR as an animal model of ADHD.

Criticism has been demonstrated specially on the predictive validity of the SHR (14, 15), which might be measured by the ability of the animal model to respond to well documented treatment for the disorder (11). In ADHD, the most well documented treatment is the one

performed with stimulant medication (16). Among stimulants, methylphenidate (MPH), a dopamine transporter inhibitor, is the most commonly used (17). Although promoting overall clinical improvement, there is a substantial heterogeneity regarding the response to MPH treatment (18), and about 30% of patients do not present a clinical response (19). Therefore, a well-validated animal model is also essential in order to have a proper tool to understand the variability in the response to MPH in a molecular level. Therefore, in this study we intend to disclose the protocols for a systematic review and meta-analysis aiming at summarizing the MPH in the SHR. With this study we aim to answer the following question: does MPH treatment improve behavioral deficits presented by the SHR when compared to placebo?

2. Methods

Methods are described according to the format proposed by de Vries, Hooijmans (20). This protocol has been previously submitted for the CAMARADES Preclinical Systematic Review & Meta-analysis Facility (SyRF, http://syrf.org.uk/protocols/) with the title "Behavioral effects of methylphenidate in an animal model of attention-deficit/hyperactivity disorder, the spontaneously hypertensive rats: a systematic review and meta-analysis".

2.1. Search and study identification

We are going to include studies that administered MPH to SHR and evaluated one of the following behavioral outcomes: locomotion, attention, impulsivity or memory. Studies will be identified through a literature search using three different electronic databases: Medline, Scopus and Web of Science. The search strategy is going to be the following: (("SHR" OR "spontaneous* hypertensive rat*") AND ("MPH" OR "methylphenidate" OR "ritalin")). No search filters are going to be used. In addition, we are going to search the reference list of included studies.

2.2. Study selection

The study selection and inclusion is going to be performed with a pre-screening based on title and abstract, followed by a full text screening if necessary. Both will be conducted by two independent authors, and any disagreement will be discussed with a third author. There will be no date or language restrictions. The following steps will be conducted for the inclusion and exclusion of studies:

Step 1: All title and abstracts are going to be reviewed by two independent authors. Those reporting experimental studies evaluating the effects of MPH in the SHR are going to be selected for the next step. The following exclusion criteria will be applied: use of SHR substrains (e.g., stroke-prone SHR), MPH administered in brain slices, MPH administered only together with another drug, MPH self-administration. If the information is not sufficient to include or exclude the study, a full-text review will be conducted;

Step 2: Studies presenting behavioral outcomes are going to be selected for the next step. The behavioral outcomes are going to be categorized according to the author´s description. Studies with no behavioral outcomes will be excluded;

Step 3: Studies reporting locomotion, attention, impulsivity or memory outcomes are going to be included for final analysis. The following exclusion criteria will be applied: distinct behavioral outcomes from the ones previously presented, use of a crossover approach, not comparing the SHR-MPH to a SHR-placebo group. Studies using a crossover approach, meaning that the same rats will be used as active and placebo groups, will be excluded in order to avoid a carryover effect (21).

2.3. Study characteristics to be extracted

For the assessment of external validity, the following items will be extracted: study authors, year of publication, experimental groups, sample size, age and gender of animals, route of drug administration, MPH dosage in mg/kg, number of administrations per day, total days of treatment, behavioral test used, and outcome of interest.

2.4. Risk of bias assessment

Risk of bias assessment will be conducted by one review author using the SYRCLE´s risk of bias tool for animal studies (22). Any doubt in the assessment will be discussed with a second reviewer. Ten items will be evaluated in the quality assessment: three related to selection bias (sequence generation, baseline characteristic and allocation concealment); two related to performance bias (random housing and blinding); two related to detection bias (random outcome assessment and blinding); one related to attrition bias (incomplete outcome data); one related to reporting bias (selective outcome reporting); and one related to other sources of bias. Baseline characteristics will be comprised by information on sex and age of animals. The tenth item will address sources of bias beyond the ones covered by other domains. Each study will be evaluated considering the ten items, and for each item the study will be classified as presenting low, unclear or high risk of bias. Publication bias will be accessed using funnel plots and the Egger´s regression test.

2.5. Collection of outcome data

Continuous outcome data from behavioral tests will be extracted by two independent authors, an any disagreement will be discussed with a third author. Data will be extracted directly from the full-text article. When not reported in enough details, extraction will be done by graph estimation using a digital ruler, as previously described (23, 24). If both methods are not viable, the authors will be contacted. Two attempt will be performed within a two-week

interval. If no response is obtained, the article will be excluded. Each included article will be divided in experiments, defined as any case when a control group is compared to an experimental group. If the same animals were subjected to more than one behavioral experiment from the same category, only one will be included (either the most relevant or the first one to be performed). In addition, only one outcome will be extracted from each behavioral experiment. Whenever the article report multiple outcomes from the same behavioral test, the extraction will be performed according to a relevance rank organized by one reviewer considering the manuscript description. If the same animals were evaluated more than once in the same behavioral test, the last one will be selected for data extraction. If the manuscript divides the results by time, the first time point will be selected for extraction.

2.6. Data analysis

2.6.1. Meta-analysis

Studies will be grouped according to the behavioral outcomes reported (locomotion, attention, impulsivity or memory), and a meta-analysis will be performed for each group. Pooled effect sizes will be determined with standardized mean differences using Hedge´s G method with random-effects, allowing the comparison among distinct behavioral tests. The significance of pooled effect sizes will be determined using the Z-test. Since we will perform four independent meta-analyses, a Bonferroni correction will be applied, and a p-value $\leq$ 0.0125 will be considered as statistically significant. Individual study weights will be obtained using the inverse of the variance. Data will be transformed in order to obtain positive values for decreased locomotion or impulsive behavior and for increased attentional or memory performances.

Heterogeneity between studies will be assessed using both the Chi^2 and the I^2 tests, and a p-value ≤ 0.1 will be considered as statistically significant. A I^2 value on the order of 25%,

50% and 75% will be considered as low, moderate, and high heterogeneity, respectively (25). Standardized mean differences and heterogeneity values will be obtained using Review Manager (RevMan) version 5.3 (Copenhagen: The Nordic Cochrane Centre, The Cochrane Collaboration, 2014).

2.6.2. Meta-regression

A meta-regression will be conducted in order to evaluate potential sources of variability among studies. The following covariates will be selected based on biological plausibility and added to a random-effects meta-regression model: age of animals; route of drug administration; MPH total dosage. Age of animals will be extracted and transformed in “days” to be added as a continuous variable in the model. Route of drug administration will be added as a categorical variable using dummy variables. MPH total dosage will be calculated by multiplying the dosage received in mg/kg by the number of administrations per day and by the total days of treatment and added as a continuous variable. Covariates associated with the outcome with a $p \leq 0.1$ in a univariate analysis will be included in a final multivariate meta-regression model, in which an alpha of 5% will be established. Studies with missing values will be excluded from the meta-regression analysis. Meta-regression will be conducted using Stata 13.0 (College Station, TX: StataCorp LP), as previously described (26).

2.6.3. Sensitivity analysis

Sensitivity analyses will be performed in order to evaluate effect size differences related to main methodological decisions in our manuscript. The following sensitivity analyses will be performed for each behavioral category: (1) excluding one result at a time; (2) including only effect sizes extracted from the same behavioral test; (3) including studies with crossover designs; (4) including only one MPH dosage at a time, with a minimum of three studies using

the same dosage; (5) excluding studies presenting a concerning risk of bias, defined as either a high risk of bias in one category or an unclear risk of bias in 7 categories or more.

The objectives of our sensitivity analyses will be to (1) observe if any study skews the overall result; (2) evaluate if the effects of MPH are different for distinct behavioral tests; (3) evaluate possible changes in effect sizes due to a carryover effect from crossover studies; (4) observe if the effects of MPH are different for distinct dosages; (4) evaluate the impact of studies with a high risk of bias. Sensitivity analyses will not be corrected for multiple comparison, and the results will be interpreted as exploratory. A p-value ≤ 0.05 will be considered statistically significant for these analyses.

3. Discussion

The pathophysiology of ADHD is still mostly unknown, and a valid animal model is believed to be essential in order to advance this knowledge. Although the SRH are the most widely used animal model of ADHD, its validity has been questioned by different authors. The poor methodological description, high heterogeneity, and high probability of bias observed in preclinical research seems to be a relevant factor for the interpretation of the published literature (27). In this sense, an overall estimate of the available data obtained using systematic reviews and meta-analyses have become more important over the years. Although they have been already used in the last decades in order to guide clinical practice, systematic reviews and meta-analyses are relatively new in preclinical research (28).

The predictive validity represents the ability of the animal model to respond to well documented treatments for the disorder, and is essential for the characterization of a valid animal model (11). In the SHR, distinct behavioral responses have been reported after a treatment with MPH. For instance, van den Bergh, Bloemarts (14) reported no effects of MPH in the animal model of ADHD, while Tamburella, Micale (29) reported increased locomotion

after MPH administration. A deleterious effect of MPH in attentional performance of SHR has also been demonstrated (30). On the other hand, these results are contradicted by several studies showing decreased locomotion (31-34) and increased attention (31, 35-37) after treatment. Even though these divergences might be explained by methodological aspects including MPH dosage, route of drug administration and age of animals, no study has been conducted in order to evaluate this hypothesis.

By conducting this study, we expect to: (1) provide an overall estimate of the effect size of MPH on behavioral outcomes when administered to the SHR; (2) provide an estimate of the heterogeneity present in the published literature; and (3) identify covariates potentially related to this heterogeneity using meta-regression and sensitivity analyses. Our main hypothesis are: (1) MPH treatment will decrease locomotion and impulsivity, and increase attention and memory performance of the SHR; (2) a high heterogeneity will be found; (3) age of animals, route of drug administration and MPH total dosage are going to be statistically related to the effects.

To sum up, we believe that the results obtained from this study will be valuable in order to corroborate the predictive validity of SHR as an animal model of ADHD. Additionally, our results will provide data on the methodological strengths of published literature, which may aid the development of new experimental designs. In this way, our study will provide a background for the development of new research and for the advance of knowledge in the area.

4. References

1. Faraone SV, Asherson P, Banaschewski T, Biederman J, Buitelaar JK, Ramos-Quiroga JA, et al. Attention-deficit/hyperactivity disorder. Nature reviews Disease primers. 2015;1:15020.
2. Danckaerts M, Sonuga-Barke EJ, Banaschewski T, Buitelaar J, Dopfner M, Hollis C, et al. The quality of life of children with attention deficit/hyperactivity disorder: a systematic review. European child & adolescent psychiatry. 2010;19(2):83-105.
3. Man KKC, Coghill D, Chan EW, Lau WCY, Hollis C, Liddle E, et al. Association of Risk of Suicide Attempts With Methylphenidate Treatment. JAMA psychiatry. 2017;74(10):1048-55.
4. Biederman J, Faraone SV. The effects of attention-deficit/hyperactivity disorder on employment and household income. MedGenMed : Medscape general medicine. 2006;8(3):12.
5. Dalsgaard S, Ostergaard SD, Leckman JF, Mortensen PB, Pedersen MG. Mortality in children, adolescents, and adults with attention deficit hyperactivity disorder: a nationwide cohort study. Lancet (London, England). 2015;385(9983):2190-6.
6. Geschwind DH, Flint J. Genetics and genomics of psychiatric disease. Science (New York, NY). 2015;349(6255):1489-94.
7. Thapar A, Cooper M. Attention deficit hyperactivity disorder. Lancet (London, England). 2016;387(10024):1240-50.
8. Hoogman M, Bralten J, Hibar DP, Mennes M, Zwiers MP, Schweren LSJ, et al. Subcortical brain volume differences in participants with attention deficit hyperactivity disorder in children and adults: a cross-sectional mega-analysis. The lancet Psychiatry. 2017;4(4):310-9.
9. Hart H, Radua J, Nakao T, Mataix-Cols D, Rubia K. Meta-analysis of functional magnetic resonance imaging studies of inhibition and attention in attention-deficit/hyperactivity disorder: exploring task-specific, stimulant medication, and age effects. JAMA psychiatry. 2013;70(2):185-98.
10. Nestler EJ, Hyman SE. Animal models of neuropsychiatric disorders. Nature neuroscience. 2010;13(10):1161-9.
11. van der Staay FJ, Arndt SS, Nordquist RE. Evaluation of animal models of neurobehavioral disorders. Behavioral and brain functions : BBF. 2009;5:11.
12. Sagvolden T, Russell VA, Aase H, Johansen EB, Farshbaf M. Rodent models of attention-deficit/hyperactivity disorder. Biological psychiatry. 2005;57(11):1239-47.
13. Russell VA. Overview of animal models of attention deficit hyperactivity disorder (ADHD). Current protocols in neuroscience. 2011;Chapter 9:Unit9.35.
14. van den Bergh FS, Bloemarts E, Chan JS, Groenink L, Olivier B, Oosting RS. Spontaneously hypertensive rats do not predict symptoms of attention-deficit hyperactivity disorder. Pharmacology, biochemistry, and behavior. 2006;83(3):380-90.
15. Wickens JR, Hyland BI, Tripp G. Animal models to guide clinical drug development in ADHD: lost in translation? British journal of pharmacology. 2011;164(4):1107-28.
16. Faraone SV, Biederman J, Spencer TJ, Aleardi M. Comparing the efficacy of medications for ADHD using meta-analysis. MedGenMed : Medscape general medicine. 2006;8(4):4.
17. Shier AC, Reichenbacher T, Ghuman HS, Ghuman JK. Pharmacological treatment of attention deficit hyperactivity disorder in children and adolescents: clinical strategies. Journal of central nervous system disease. 2013;5:1-17.

18. Wilens TE, Morrison NR, Prince J. An update on the pharmacotherapy of attention-deficit/hyperactivity disorder in adults. Expert Rev Neurother. 2011;11(10):1443-65.
19. Pliszka SR. Pharmacologic treatment of attention-deficit/hyperactivity disorder: efficacy, safety and mechanisms of action. Neuropsychology review. 2007;17(1):61-72.
20. de Vries RBM, Hooijmans CR, Langendam MW, van Luijk J, Leenaars M, Ritskes-Hoitinga M, et al. A protocol format for the preparation, registration and publication of systematic reviews of animal intervention studies. Evidence-based Preclinical Medicine. 2015;2(1):1-9.
21. Curtin F, Elbourne D, Altman DG. Meta-analysis combining parallel and cross-over clinical trials. III: The issue of carry-over. Statistics in medicine. 2002;21(15):2161-73.
22. Hooijmans CR, Rovers MM, de Vries RB, Leenaars M, Ritskes-Hoitinga M, Langendam MW. SYRCLE's risk of bias tool for animal studies. BMC medical research methodology. 2014;14:43.
23. Pires GN, Bezerra AG, Tufik S, Andersen ML. Effects of experimental sleep deprivation on anxiety-like behavior in animal research: Systematic review and meta-analysis. Neuroscience and biobehavioral reviews. 2016;68:575-89.
24. Pires GN, Bezerra AG, Tufik S, Andersen ML. Effects of acute sleep deprivation on state anxiety levels: a systematic review and meta-analysis. Sleep medicine. 2016;24:109-18.
25. Higgins JP, Thompson SG. Quantifying heterogeneity in a meta-analysis. Statistics in medicine. 2002;21(11):1539-58.
26. Harbord RM, Higgins JPT. Meta-regression in Stata. Stata Journal. 2008;8(4):493-519.
27. Kilkenny C, Parsons N, Kadyszewski E, Festing MF, Cuthill IC, Fry D, et al. Survey of the quality of experimental design, statistical analysis and reporting of research using animals. PloS one. 2009;4(11):e7824.
28. Sena ES, Currie GL, McCann SK, Macleod MR, Howells DW. Systematic reviews and meta-analysis of preclinical studies: why perform them and how to appraise them critically. Journal of cerebral blood flow and metabolism : official journal of the International Society of Cerebral Blood Flow and Metabolism. 2014;34(5):737-42.
29. Tamburella A, Micale V, Mazzola C, Salomone S, Drago F. The selective norepinephrine reuptake inhibitor atomoxetine counteracts behavioral impairments in trimethyltin-intoxicated rats. European journal of pharmacology. 2012;683(1-3):148-54.
30. Aspide R, Fresiello A, de Filippis G, Gironi Carnevale UA, Sadile AG. Non-selective attention in a rat model of hyperactivity and attention deficit: subchronic methylphenydate and nitric oxide synthesis inhibitor treatment. Neuroscience and biobehavioral reviews. 2000;24(1):59-71.
31. Hong Q, Zhang M, Pan XQ, Guo M, Li F, Tong ML, et al. Prefrontal cortex Homer expression in an animal model of attention-deficit/hyperactivity disorder. Journal of the neurological sciences. 2009;287(1-2):205-11.
32. Somkuwar SS, Kantak KM, Bardo MT, Dwoskin LP. Adolescent methylphenidate treatment differentially alters adult impulsivity and hyperactivity in the Spontaneously Hypertensive Rat model of ADHD. Pharmacology, biochemistry, and behavior. 2016;141:66-77.
33. Kim H, Heo HI, Kim DH, Ko IG, Lee SS, Kim SE, et al. Treadmill exercise and methylphenidate ameliorate symptoms of attention deficit/hyperactivity disorder through enhancing dopamine synthesis and brain-derived neurotrophic factor expression in spontaneous hypertensive rats. Neuroscience letters. 2011;504(1):35-9.

34. Yang MT, Lu DH, Chen JC, Fu WM. Inhibition of hyperactivity and impulsivity by carbonic anhydrase inhibitors in spontaneously hypertensive rats, an animal model of ADHD. Psychopharmacology. 2015;232(20):3763-72.
35. Cheng JT, Li JS. Intra-orbitofrontal cortex injection of haloperidol removes the beneficial effect of methylphenidate on reversal learning of spontaneously hypertensive rats in an attentional set-shifting task. Behavioural brain research. 2013;239:148-54.
36. Fox GB, Pan JB, Esbenshade TA, Bennani YL, Black LA, Faghih R, et al. Effects of histamine H(3) receptor ligands GT-2331 and ciproxifan in a repeated acquisition avoidance response in the spontaneously hypertensive rat pup. Behavioural brain research. 2002;131(1-2):151-61.
37. Robinson AM, Eggleston RL, Bucci DJ. Physical exercise and catecholamine reuptake inhibitors affect orienting behavior and social interaction in a rat model of attention-deficit/hyperactivity disorder. Behavioral neuroscience. 2012;126(6):762-71.

Behavioral effects of methylphenidate in an animal model of attention-deficit/hyperactivity disorder, the spontaneously hypertensive rats: a systematic review and meta-analysis protocol

Douglas Teixeira Leffa[1,2], Alana Castro Panzenhagen[3,4], Diego Luiz Rovaris[3,4,5], Iraci L.S. Torres[1,2], Luis Augusto Rohde[4,5,6], Eugenio Horacio Grevet[4,5], Gabriel Natan Pires[7]

[1]Post-Graduate Program in Medicine: Medical Sciences, School of Medicine, Universidade Federal do Rio Grande do Sul, Porto Alegre, Brazil

[2]Laboratory of Pain Pharmacology and Neuromodulation: Pre clinical studies - Pharmacology Department, Institute of Basic Health Sciences, Universidade Federal do Rio Grande do Sul, Porto Alegre, Brazil

[3]Department of Genetics, Universidade Federal do Rio Grande do Sul, Porto Alegre, Brazil.

[4]ADHD Outpatient Program, Hospital de Clínicas de Porto Alegre, Brazil

[5]Department of Psychiatry, Universidade Federal do Rio Grande do Sul, Porto Alegre, Brazil

[6]National Institute of Developmental Psychiatry for Children and Adolescents, Brazil

[7]Departamento de Psicobiologia, Universidade Federal de São Paulo – São Paulo, Brazil

Corresponding author:

Douglas Teixeira Leffa

Post-Graduate Program in Medicine: Medical Sciences - School of Medicine, UFRGS

Rua Ramiro Barcelos – 2400

90035-003 – Porto Alegre, RS, Brazil

Phone: (55-51)3308.5232

E-mail: douglasleffa@hotmail.com

Abstract

Attention-deficit/hyperactivity disorder (ADHD) is a prevalent condition related to several negative outcomes. Although a strong neurobiological basis has been demonstrated, its pathophysiology is still poorly understood. In this sense, animal studies are essential in order to unravel the neurobiological factors associated with neuropsychiatric disorders, and the absence of proper animal models may be contributing to the currently challenges in the development of new treatments. The spontaneously hypertensive rats (SHR) are the most commonly used animal model of ADHD. However, its validity, and especially its predictive validity, has been questioned. In this study we aim at summarizing the evidence for the predictive validity of the SHR as an animal model of ADHD. The current protocol discloses the background, aims and methods of a systematic review and meta-analysis of studies reporting behavioral effects of methylphenidate (MPH), the most commonly prescribed treatment for ADHD, in the SHR. In addition, we plan to evaluate how pre-defined covariates influence the effects of MPH using meta-regression and sensitivity analyses.

Keywords: ADHD, SHR, meta-analysis, methylphenidate

1. Introduction

Attention-deficit/hyperactivity disorder (ADHD) is a prevalent neurodevelopmental disorder characterized by impairing levels of hyperactivity, impulsivity and inattention (1). Observational studies have shown that ADHD is related to several negative outcomes, including, among others, decreased quality of life (2), increased number of suicide attempts (3), less socioeconomic status (4), and increased mortality due to accidents (5).

A strong neurobiological basis has been repeatedly demonstrated in ADHD. Twin studies showed that ADHD has a heritability of about 70-80% (6), and candidate gene studies suggest mainly the influence of genes related to monoaminergic neurotransmission (7). A recent mega-analysis performed with more than 1500 ADHD patients found a reduced volume in the nucleus accumbens, amygdala, caudate, hippocampus and putamen in patients with the disorder (8). In addition, a meta-analysis of fMRI studies demonstrated reduced activation in distinct cortical region during attention and impulsivity tests (9). Although ADHD neurobiological basis has been confirmed through molecular genetics and neuroimaging studies, its pathophysiology is still poorly understood.

Animal models are considered a fundamental tool to unravel the neurobiological factors associated with neuropsychiatric disorders (10). More important, the absence of proper animal models has been proposed as a main factor driving the slow advancement of new treatments for neuropsychiatric disorders (10). An animal model should present predictive, face and construct validity in order to be considered a proper model of a disease (11). In ADHD, the spontaneously hypertensive rats (SHR) are widely considered the most appropriate model (12, 13). However, there is no consensus on the validity of the SHR as an animal model of ADHD.

Criticism has been demonstrated specially on the predictive validity of the SHR (14, 15), which might be measured by the ability of the animal model to respond to well documented treatment for the disorder (11). In ADHD, the most well documented treatment is the one

performed with stimulant medication (16). Among stimulants, methylphenidate (MPH), a dopamine transporter inhibitor, is the most commonly used (17). Although promoting overall clinical improvement, there is a substantial heterogeneity regarding the response to MPH treatment (18), and about 30% of patients do not present a clinical response (19). Therefore, a well-validated animal model is also essential in order to have a proper tool to understand the variability in the response to MPH in a molecular level. Thus, in this study we intend to disclose the protocols for a systematic review and meta-analysis aiming at summarizing behavioral effects of MPH in the SHR. With this study we aim to answer the following question: does MPH treatment improve behavioral deficits presented by the SHR when compared to placebo? In order to achieve this aim, the following objectives were defined: (1) conduct an extensive literature research in order to select all studies evaluating behavioral effects of MPH in the SHR; (2) extract data; (3) summarize the data using meta-analysis; and (4) explore the influence of pre-defined variables using meta-regression and sensitivity analysis.

2. Methods

Methods are described according to the format proposed by de Vries, Hooijmans (20). This protocol has been previously submitted for the CAMARADES Preclinical Systematic Review & Meta-analysis Facility (SyRF, http://syrf.org.uk/protocols/) with the title "Behavioral effects of methylphenidate in an animal model of attention-deficit/hyperactivity disorder, the spontaneously hypertensive rats: a systematic review and meta-analysis".

2.1. Search and study identification

We are going to include studies that administered MPH to SHR and evaluated one of the following behavioral outcomes: locomotion, attention, impulsivity or memory. Studies will be identified through a literature search using three different electronic databases: Medline,

Scopus and Web of Science. The complete search strategy can be found in Supplementary material 1. No search filters are going to be used. In addition, we are going to search the reference list of included studies.

2.2. Study selection

The study selection and inclusion is going to be performed with a pre-screening based on title and abstract. If an exclusion criteria is not clearly observed (e.g., reviews or editorials, studies in human subjects), a full-text review will be performed. Both will be conducted by two independent authors (D.L. and A.P.), and any disagreement will be discussed with a third author (E.G.). There will be no date or language restrictions. The following steps will be conducted for the inclusion and exclusion of studies:

Step 1: All title and abstracts are going to be reviewed by two independent authors (D.L. and A.P.). Those reporting experimental studies evaluating the effects of MPH in the SHR are going to be selected for the next step. The following exclusion criteria will be applied: use of SHR substrains (e.g., stroke-prone SHR), MPH administered in brain slices, MPH administered only together with another drug, MPH self-administration. If the information is not sufficient to exclude the study, a full-text review will be conducted;

Step 2: Studies presenting behavioral outcomes are going to be selected for the next step. The behavioral outcomes are going to be categorized according to the author´s description. Studies with no behavioral outcomes will be excluded;

Step 3: Studies reporting locomotion, attention, impulsivity or memory outcomes are going to be included for final analysis. The following exclusion criteria will be applied: distinct behavioral outcomes from the ones previously presented, use of a crossover approach, not comparing the SHR-MPH to a SHR-placebo group. Studies using a crossover approach,

meaning that the same rats will be used as active and placebo groups, will be excluded in order to avoid a carryover effect (21).

2.3. Study characteristics to be extracted

For the assessment of external validity, the following items will be extracted: study authors, year of publication, experimental groups, sample size, age and gender of animals, route of drug administration, MPH dosage in mg/kg, number of administrations per day, total days of treatment, behavioral test used, and outcome of interest.

2.4. Risk of bias assessment

Risk of bias assessment will be conducted by one review (A.P.) author using the SYRCLE´s risk of bias tool for animal studies (22). Any doubt in the assessment will be discussed with a second reviewer (D.L.). Ten items will be evaluated in the quality assessment: three related to selection bias (sequence generation, baseline characteristic and allocation concealment); two related to performance bias (random housing and blinding); two related to detection bias (random outcome assessment and blinding); one related to attrition bias (incomplete outcome data); one related to reporting bias (selective outcome reporting); and one related to other sources of bias. Baseline characteristics will be comprised by information on sex and age of animals. The tenth item will address sources of bias beyond the ones covered by other domains. Each study will be evaluated considering the ten items, and for each item the study will be classified as presenting low, unclear or high risk of bias. Publication bias will be accessed using funnel plots and the Egger´s regression test.

2.5. Collection of outcome data

Continuous outcome data from behavioral tests will be extracted by two independent authors (D.L. and A.P.), an any disagreement will be discussed with a third author (E.G.). Data will be extracted directly from the full-text article. When not reported in enough details, extraction will be done by graph estimation using a digital ruler, as previously described (23, 24). If both methods are not viable, the authors will be contacted. Two attempt will be performed within a two-week interval. If no response is obtained, the article will be excluded. Each included article will be divided in experiments, defined as any case when a control group is compared to an experimental group. If the same animals were subjected to more than one behavioral experiment from the same category, only one will be included (either the most relevant or the first one to be performed). In addition, only one outcome will be extracted from each behavioral experiment. Whenever the article report multiple outcomes from the same behavioral test, the extraction will be performed according to a relevance rank organized by one reviewer. Variables from each behavioral test will be ranked subjectively according to their importance, and the one ranked highest will be extracted. If the same animals were evaluated more than once in the same behavioral test, the last one will be selected for data extraction. If the manuscript divides the results by time, the first time point will be selected for extraction.

2.6. Data analysis

2.6.1. Meta-analysis

Studies will be grouped according to the behavioral outcomes reported (locomotion, attention, impulsivity or memory), and a meta-analysis will be performed for each group. Pooled effect sizes will be determined with standardized mean differences using Hedge´s G method with random-effects, allowing the comparison among distinct behavioral tests. The significance of pooled effect sizes will be determined using the Z-test. Since we will perform four independent meta-analyses, a Bonferroni correction will be applied, and a p-value ≤

0.0125 will be considered as statistically significant. Individual study weights will be obtained using the inverse of the variance. Data will be transformed in order to obtain positive values for decreased locomotion or impulsive behavior and for increased attentional or memory performances.

Heterogeneity between studies will be assessed using both the Chi^2 and the I^2 tests, and a p-value ≤ 0.1 will be considered as statistically significant. A I^2 value on the order of 25%, 50% and 75% will be considered as low, moderate, and high heterogeneity, respectively (25). Standardized mean differences and heterogeneity values will be obtained using Review Manager (RevMan) version 5.3 (Copenhagen: The Nordic Cochrane Centre, The Cochrane Collaboration, 2014).

2.6.2. Meta-regression

A meta-regression will be conducted in order to evaluate potential sources of variability among studies. The following covariates will be selected based on biological plausibility and added to a random-effects meta-regression model: age of animals; route of drug administration; MPH total dosage. Age of animals will be extracted and transformed in “days” to be added as a continuous variable in the model. Route of drug administration will be added as a categorical variable using dummy variables. MPH total dosage will be calculated by multiplying the dosage received in mg/kg by the number of administrations per day and by the total days of treatment and added as a continuous variable. Covariates associated with the outcome with a $p \leq 0.1$ in a univariate analysis will be included in a final multivariate meta-regression model, in which an alpha of 5% will be established. Studies with missing values will be excluded from the meta-regression analysis. Meta-regression will be conducted using Stata 13.0 (College Station, TX: StataCorp LP), as previously described (26).

2.6.3. Sensitivity analysis

Sensitivity analyses will be performed in order to evaluate effect size differences related to main methodological decisions in our manuscript. The following sensitivity analyses will be performed for each behavioral category: (1) the jackknife method, a common procedure used to test the stability of the outcome after excluding one result at a time (27); (2) including only effect sizes extracted from the same behavioral test; (3) including studies with crossover designs; (4) including only one MPH dosage at a time, with a minimum of three studies using the same dosage; (5) excluding studies presenting a concerning risk of bias, defined as either a high risk of bias in one category or an unclear risk of bias in 7 categories or more.

The objectives of our sensitivity analyses will be to (1) observe if any study skews the overall result; (2) evaluate if the effects of MPH are different for distinct behavioral tests; (3) evaluate possible changes in effect sizes due to a carryover effect from crossover studies; (4) observe if the effects of MPH are different for distinct dosages; (4) evaluate the impact of studies with a high risk of bias. Sensitivity analyses will not be corrected for multiple comparison, and the results will be interpreted as exploratory. A p-value ≤ 0.05 will be considered statistically significant for these analyses.

3. Discussion

The pathophysiology of ADHD is still mostly unknown, and a valid animal model is believed to be essential in order to advance this knowledge. Although the SRH are the most widely used animal model of ADHD, its validity has been questioned by different authors. The poor methodological description, high heterogeneity, and high probability of bias observed in preclinical research seems to be a relevant factor for the interpretation of the published literature (28). In this sense, an overall estimate of the available data obtained using systematic reviews and meta-analyses have become more important over the years. Although they have

been already used in the last decades in order to guide clinical practice, systematic reviews and meta-analyses are relatively new in preclinical research (29).

The predictive validity represents the ability of the animal model to respond to well documented treatments for the disorder, and is essential for the characterization of a valid animal model (11). In the SHR, distinct behavioral responses have been reported after a treatment with MPH. For instance, van den Bergh, Bloemarts (14) reported no effects of MPH in the animal model of ADHD, while Tamburella, Micale (30) reported increased locomotion after MPH administration. A deleterious effect of MPH in attentional performance of SHR has also been demonstrated (31). On the other hand, these results are contradicted by several studies showing decreased locomotion (32-35) and increased attention (32, 36-38) after treatment. Even though these divergences might be explained by methodological aspects including MPH dosage, route of drug administration and age of animals, no study has been conducted in order to evaluate this hypothesis.

By conducting this study, we expect to: (1) provide an overall estimate of the effect size of MPH on behavioral outcomes when administered to the SHR; (2) provide an estimate of the heterogeneity present in the published literature; and (3) identify covariates potentially related to this heterogeneity using meta-regression and sensitivity analyses. Our main hypothesis are: (1) MPH treatment will decrease locomotion and impulsivity, and increase attention and memory performance of the SHR; (2) a high heterogeneity will be found; (3) age of animals, route of drug administration and MPH total dosage are going to be statistically related to the effects.

A detailed description of the methodology to be applied in systematic reviews and meta-analyses has many advantages, including expert external opinion on the methods, prevention of data fishing, and the possibility of helping researchers in future applications. Additionally, protocol publication is an opportunity to discuss potential sources of bias that

may be present in the future. In this sense, the following factors should be highlighted from our methodology. First, we are going to include only one data point for each behavioral test, which will be selected based on a subjective relevance rank. Although distinct approaches were available for the extraction (e.g. the combination of multiple variables into a single data point), we believe that the use of only one variable may ensure increased consistency among results. It is known that the same behavioral test can be performed in order to investigate distinct behavioral parameters. As an example, the open field test can be used to measure locomotion (39), anxiety-related behavior (40), or spatial memory (41), based primarily on which variable is collected. Therefore, even though our approach may present a risk of bias for being based on a subjective evaluation of importance, we believe that the grouping of multiple variables into a single data point would be an even greater threat since it would combine distinct information, thus increasing heterogeneity. Another important aspect is related to our meta-regression model. As previously mentioned, studies with missing values will be excluded from the analysis, which can bias the final results.

To sum up, we believe that the results obtained from this study will be valuable in order to corroborate the predictive validity of SHR as an animal model of ADHD. Additionally, our results will provide data on the methodological strengths of published literature, which may aid the development of new experimental designs. In this way, our study will provide a background for the development of new research and for the advance of knowledge in the area.

4. References

1. Faraone SV, Asherson P, Banaschewski T, Biederman J, Buitelaar JK, Ramos-Quiroga JA, et al. Attention-deficit/hyperactivity disorder. Nature reviews Disease primers. 2015;1:15020.
2. Danckaerts M, Sonuga-Barke EJ, Banaschewski T, Buitelaar J, Dopfner M, Hollis C, et al. The quality of life of children with attention deficit/hyperactivity disorder: a systematic review. European child & adolescent psychiatry. 2010;19(2):83-105.
3. Man KKC, Coghill D, Chan EW, Lau WCY, Hollis C, Liddle E, et al. Association of Risk of Suicide Attempts With Methylphenidate Treatment. JAMA psychiatry. 2017;74(10):1048-55.
4. Biederman J, Faraone SV. The effects of attention-deficit/hyperactivity disorder on employment and household income. MedGenMed : Medscape general medicine. 2006;8(3):12.
5. Dalsgaard S, Ostergaard SD, Leckman JF, Mortensen PB, Pedersen MG. Mortality in children, adolescents, and adults with attention deficit hyperactivity disorder: a nationwide cohort study. Lancet (London, England). 2015;385(9983):2190-6.
6. Geschwind DH, Flint J. Genetics and genomics of psychiatric disease. Science (New York, NY). 2015;349(6255):1489-94.
7. Thapar A, Cooper M. Attention deficit hyperactivity disorder. Lancet (London, England). 2016;387(10024):1240-50.
8. Hoogman M, Bralten J, Hibar DP, Mennes M, Zwiers MP, Schweren LSJ, et al. Subcortical brain volume differences in participants with attention deficit hyperactivity disorder in children and adults: a cross-sectional mega-analysis. The lancet Psychiatry. 2017;4(4):310-9.
9. Hart H, Radua J, Nakao T, Mataix-Cols D, Rubia K. Meta-analysis of functional magnetic resonance imaging studies of inhibition and attention in attention-deficit/hyperactivity disorder: exploring task-specific, stimulant medication, and age effects. JAMA psychiatry. 2013;70(2):185-98.
10. Nestler EJ, Hyman SE. Animal models of neuropsychiatric disorders. Nature neuroscience. 2010;13(10):1161-9.
11. van der Staay FJ, Arndt SS, Nordquist RE. Evaluation of animal models of neurobehavioral disorders. Behavioral and brain functions : BBF. 2009;5:11.
12. Sagvolden T, Russell VA, Aase H, Johansen EB, Farshbaf M. Rodent models of attention-deficit/hyperactivity disorder. Biological psychiatry. 2005;57(11):1239-47.
13. Russell VA. Overview of animal models of attention deficit hyperactivity disorder (ADHD). Current protocols in neuroscience. 2011;Chapter 9:Unit9.35.
14. van den Bergh FS, Bloemarts E, Chan JS, Groenink L, Olivier B, Oosting RS. Spontaneously hypertensive rats do not predict symptoms of attention-deficit hyperactivity disorder. Pharmacology, biochemistry, and behavior. 2006;83(3):380-90.
15. Wickens JR, Hyland BI, Tripp G. Animal models to guide clinical drug development in ADHD: lost in translation? British journal of pharmacology. 2011;164(4):1107-28.
16. Faraone SV, Biederman J, Spencer TJ, Aleardi M. Comparing the efficacy of medications for ADHD using meta-analysis. MedGenMed : Medscape general medicine. 2006;8(4):4.
17. Shier AC, Reichenbacher T, Ghuman HS, Ghuman JK. Pharmacological treatment of attention deficit hyperactivity disorder in children and adolescents: clinical strategies. Journal of central nervous system disease. 2013;5:1-17.

18. Wilens TE, Morrison NR, Prince J. An update on the pharmacotherapy of attention-deficit/hyperactivity disorder in adults. Expert Rev Neurother. 2011;11(10):1443-65.
19. Pliszka SR. Pharmacologic treatment of attention-deficit/hyperactivity disorder: efficacy, safety and mechanisms of action. Neuropsychology review. 2007;17(1):61-72.
20. de Vries RBM, Hooijmans CR, Langendam MW, van Luijk J, Leenaars M, Ritskes-Hoitinga M, et al. A protocol format for the preparation, registration and publication of systematic reviews of animal intervention studies. Evidence-based Preclinical Medicine. 2015;2(1):1-9.
21. Curtin F, Elbourne D, Altman DG. Meta-analysis combining parallel and cross-over clinical trials. III: The issue of carry-over. Statistics in medicine. 2002;21(15):2161-73.
22. Hooijmans CR, Rovers MM, de Vries RB, Leenaars M, Ritskes-Hoitinga M, Langendam MW. SYRCLE's risk of bias tool for animal studies. BMC medical research methodology. 2014;14:43.
23. Pires GN, Bezerra AG, Tufik S, Andersen ML. Effects of experimental sleep deprivation on anxiety-like behavior in animal research: Systematic review and meta-analysis. Neuroscience and biobehavioral reviews. 2016;68:575-89.
24. Pires GN, Bezerra AG, Tufik S, Andersen ML. Effects of acute sleep deprivation on state anxiety levels: a systematic review and meta-analysis. Sleep medicine. 2016;24:109-18.
25. Higgins JP, Thompson SG. Quantifying heterogeneity in a meta-analysis. Statistics in medicine. 2002;21(11):1539-58.
26. Harbord RM, Higgins JPT. Meta-regression in Stata. Stata Journal. 2008;8(4):493-519.
27. Miller RG. The jackknife-a review. Biometrika. 1974;61(1):1-15.
28. Kilkenny C, Parsons N, Kadyszewski E, Festing MF, Cuthill IC, Fry D, et al. Survey of the quality of experimental design, statistical analysis and reporting of research using animals. PloS one. 2009;4(11):e7824.
29. Sena ES, Currie GL, McCann SK, Macleod MR, Howells DW. Systematic reviews and meta-analysis of preclinical studies: why perform them and how to appraise them critically. Journal of cerebral blood flow and metabolism : official journal of the International Society of Cerebral Blood Flow and Metabolism. 2014;34(5):737-42.
30. Tamburella A, Micale V, Mazzola C, Salomone S, Drago F. The selective norepinephrine reuptake inhibitor atomoxetine counteracts behavioral impairments in trimethyltin-intoxicated rats. European journal of pharmacology. 2012;683(1-3):148-54.
31. Aspide R, Fresiello A, de Filippis G, Gironi Carnevale UA, Sadile AG. Non-selective attention in a rat model of hyperactivity and attention deficit: subchronic methylphenydate and nitric oxide synthesis inhibitor treatment. Neuroscience and biobehavioral reviews. 2000;24(1):59-71.
32. Hong Q, Zhang M, Pan XQ, Guo M, Li F, Tong ML, et al. Prefrontal cortex Homer expression in an animal model of attention-deficit/hyperactivity disorder. Journal of the neurological sciences. 2009;287(1-2):205-11.
33. Somkuwar SS, Kantak KM, Bardo MT, Dwoskin LP. Adolescent methylphenidate treatment differentially alters adult impulsivity and hyperactivity in the Spontaneously Hypertensive Rat model of ADHD. Pharmacology, biochemistry, and behavior. 2016;141:66-77.
34. Kim H, Heo HI, Kim DH, Ko IG, Lee SS, Kim SE, et al. Treadmill exercise and methylphenidate ameliorate symptoms of attention deficit/hyperactivity disorder through enhancing dopamine synthesis and brain-derived neurotrophic factor expression in spontaneous hypertensive rats. Neuroscience letters. 2011;504(1):35-9.

35. Yang MT, Lu DH, Chen JC, Fu WM. Inhibition of hyperactivity and impulsivity by carbonic anhydrase inhibitors in spontaneously hypertensive rats, an animal model of ADHD. Psychopharmacology. 2015;232(20):3763-72.
36. Cheng JT, Li JS. Intra-orbitofrontal cortex injection of haloperidol removes the beneficial effect of methylphenidate on reversal learning of spontaneously hypertensive rats in an attentional set-shifting task. Behavioural brain research. 2013;239:148-54.
37. Fox GB, Pan JB, Esbenshade TA, Bennani YL, Black LA, Faghih R, et al. Effects of histamine H(3) receptor ligands GT-2331 and ciproxifan in a repeated acquisition avoidance response in the spontaneously hypertensive rat pup. Behavioural brain research. 2002;131(1-2):151-61.
38. Robinson AM, Eggleston RL, Bucci DJ. Physical exercise and catecholamine reuptake inhibitors affect orienting behavior and social interaction in a rat model of attention-deficit/hyperactivity disorder. Behavioral neuroscience. 2012;126(6):762-71.
39. Tatem KS, Quinn JL, Phadke A, Yu Q, Gordish-Dressman H, Nagaraju K. Behavioral and Locomotor Measurements Using an Open Field Activity Monitoring System for Skeletal Muscle Diseases. Journal of Visualized Experiments : JoVE. 2014(91).
40. Lecorps B, Rodel HG, Feron C. Assessment of anxiety in open field and elevated plus maze using infrared thermography. Physiology & behavior. 2016;157:209-16.
41. Dvorkin A, Benjamini Y, Golani I. Mouse Cognition-Related Behavior in the Open-Field: Emergence of Places of Attraction. PLOS Computational Biology. 2008;4(2):e1000027.

Behavioral effects of methylphenidate in an animal model of attention-deficit/hyperactivity disorder, the spontaneously hypertensive rats: a systematic review and meta-analysis protocol

Douglas Teixeira Leffa[1,2], Alana Castro Panzenhagen[3,4], Diego Luiz Rovaris[3,4,5], Claiton Henrique Dotto Bau[3,4,5] Luis Augusto Rohde[4,5,6], Eugenio Horacio Grevet[4,5], Gabriel Natan Pires[7,8]

[1]Post-Graduate Program in Medicine: Medical Sciences, School of Medicine, Universidade Federal do Rio Grande do Sul, Porto Alegre, Brazil

[2]Laboratory of Pain Pharmacology and Neuromodulation: Pre clinical studies - Pharmacology Department, Institute of Basic Health Sciences, Universidade Federal do Rio Grande do Sul, Porto Alegre, Brazil

[3]Department of Genetics, Universidade Federal do Rio Grande do Sul, Porto Alegre, Brazil.

[4]ADHD Outpatient Program, Hospital de Clínicas de Porto Alegre, Brazil

[5]Department of Psychiatry, Universidade Federal do Rio Grande do Sul, Porto Alegre, Brazil

[6]National Institute of Developmental Psychiatry for Children and Adolescents, Brazil

[7]Departamento de Psicobiologia, Universidade Federal de São Paulo – São Paulo, Brazil

[8]Department of Physiological Sciences, Santa Casa de São Paulo School of Medical Sciences - São Paulo, Brazil

Corresponding author:

Douglas Teixeira Leffa

Post-Graduate Program in Medicine: Medical Sciences - School of Medicine, UFRGS

Rua Ramiro Barcelos – 2400

90035-003 – Porto Alegre, RS, Brazil

Phone: (55-51)3308.5232

E-mail: douglasleffa@hotmail.com

Abstract

Attention-deficit/hyperactivity disorder (ADHD) is a prevalent condition related to several negative outcomes. Although a strong neurobiological basis has been demonstrated, its pathophysiology is still poorly understood. In this sense, proper animal models are needed to further unravel the neurobiological basis of ADHD. The spontaneously hypertensive rats (SHR) are the most commonly used animal model of ADHD. However, its validity, and especially its predictive validity, has been questioned. The current protocol discloses the background, aims and methods of a systematic review and meta-analysis of studies reporting behavioral effects of methylphenidate (MPH), the most commonly prescribed treatment for ADHD, in the SHR. The following behavioral outcomes will be evaluated: locomotion, attention, impulsivity and memory. In addition, we plan to evaluate how pre-defined covariates influence the effects of MPH using meta-regression and sensitivity analyses.

Keywords: ADHD, SHR, meta-analysis, methylphenidate

1. Introduction

Attention-deficit/hyperactivity disorder (ADHD) is a prevalent neurodevelopmental disorder characterized by impairing levels of hyperactivity, impulsivity and inattention (1). Observational studies have shown that ADHD is related to several negative outcomes, including, among others, decreased quality of life (2), increased number of suicide attempts (3), less socioeconomic status (4), and increased mortality due to accidents (5).

A strong neurobiological basis has been repeatedly demonstrated in ADHD. Twin studies showed that ADHD has a heritability of about 70-80% (6), and significant genome-wide hits have been reported, especially in neurodevelopmental processes that are relevant to ADHD (7). A recent meta-analysis performed with more than 1500 ADHD patients found a reduced volume in the nucleus accumbens, amygdala, caudate, hippocampus and putamen in patients with the disorder (8). In addition, a meta-analysis of functional magnetic resonance imaging studies demonstrated reduced activation in distinct cortical region during attention and impulsivity tests (9). Even though molecular genetics and neuroimaging studies have been improving our understanding of the disorder, the pathophysiology of ADHD is still poorly understood.

Animal models are considered a fundamental tool to unravel the neurobiological factors associated with neuropsychiatric disorders (10). More importantly, the absence of proper animal models has been proposed as a main factor driving the slow advancement of new treatments for neuropsychiatric disorders (10). An animal model should present predictive, face and construct validity in order to be considered a proper model of a disease (11). In ADHD, the spontaneously hypertensive rats (SHR) are widely considered the most appropriate model (12, 13). Evidence has shown that the SHR present face validity (12, 13), which corresponds to the extent of similarities between the animal model and the disorder. The evaluation of construct validity, which represents the resemblance between the

etiological processes in patients and animal model, is still a challenge because the pathophysiology of ADHD is mostly unknown. In addition, there is no consensus on the predictive and construct validity of the SHR as an animal model of ADHD.

Construct validity depends on the understanding of the pathophysiology of the disorder, while predictive validity depends on the similarity between the response to treatment in both humans and animals. Criticism has been demonstrated on the predictive validity of the SHR (14, 15), which might be measured by the ability of the animal model to respond to well documented treatment for the disorder (11). In ADHD, the most well documented treatment is the one performed with stimulant medication (16). Among stimulants, methylphenidate (MPH), a dopamine transporter inhibitor, is the most commonly used (17). Although promoting overall clinical improvement, there is a substantial heterogeneity regarding the response to MPH treatment (18), and about 30% of patients do not present a clinical response (19). Therefore, a well-validated animal model is also essential in order to have a proper tool to understand the variability in the response to MPH on a molecular level. Thus, in this study we intend to disclose the protocols for a systematic review and meta-analysis aiming at summarizing behavioral effects of MPH in the SHR. With this study we aim to answer the following question: does MPH treatment improve behavioral deficits presented by the SHR when compared to placebo? In order to achieve this aim, the following objectives were defined: (1) conduct an extensive literature research in order to select all studies evaluating behavioral effects of MPH in the SHR; (2) extract data; (3) summarize the data using meta-analysis; and (4) explore the influence of pre-defined variables using meta-regression and sensitivity analysis. Since MPH is the pharmacological treatment most commonly prescribed for ADHD patients, our results will be important in order to reinforce the predictive validity of the SHR.

2. Methods

Methods are described according to the format proposed by de Vries, Hooijmans (20). This protocol has been previously submitted for the CAMARADES Preclinical Systematic Review & Meta-analysis Facility (SyRF, http://syrf.org.uk/protocols/) with the title "Behavioral effects of methylphenidate in an animal model of attention-deficit/hyperactivity disorder, the spontaneously hypertensive rats: a systematic review and meta-analysis".

2.1. Search and study identification

We are going to include studies that administered MPH to SHR and evaluated one of the following behavioral outcomes: locomotion, attention, impulsivity or memory. Studies will be identified through a literature search using three different electronic databases: Medline, Embase and Web of Science. The complete search strategy can be found in Supplementary material 1. No search filters are going to be used. In addition, we are going to search the reference list of included studies.

2.2. Study selection

The study selection and inclusion is going to be performed with a pre-screening based on title and abstract. If an exclusion criterion is not clearly observed (e.g., reviews or editorials, studies in human subjects), a full-text review will be performed. Both will be conducted by two independent authors (D.L. and A.P.), and any disagreement will be discussed with a third author (E.G.). There will be no date or language restrictions. We are going to include experimental studies evaluating the effects of MPH in the SHR in the following behavioral outcomes: locomotion, attention, impulsivity or memory. Only studies that administered MPH to SHR and have a control group will be included in the analysis. The following exclusion criteria will be applied: use of SHR substrains (e.g., stroke-prone SHR),

MPH administered in brain slices, MPH administered only together with another drug, and MPH self-administration. Studies using a crossover approach, meaning that the same rats will be used as active and placebo groups, will be excluded in order to avoid a carryover effect (21).

2.3. Study characteristics to be extracted

For the assessment of external validity, the following items will be extracted: study authors, year of publication, experimental groups, sample size, age and sex of animals, route of drug administration, MPH dosage in mg/kg, number of administrations per day, total days of treatment, behavioral test used, and outcome of interest.

2.4. Risk of bias assessment

Risk of bias assessment will be conducted by one review (A.P.) author using the SYRCLE´s risk of bias tool for animal studies (22). Any doubt in the assessment will be discussed with a second reviewer (D.L.). Ten items will be evaluated in the quality assessment: three related to selection bias (sequence generation, baseline characteristic and allocation concealment); two related to performance bias (random housing and blinding); two related to detection bias (random outcome assessment and blinding); one related to attrition bias (incomplete outcome data); one related to reporting bias (selective outcome reporting); and one related to other sources of bias. Baseline characteristics will be comprised by information on sex and age of animals. The tenth item will address sources of bias beyond the ones covered by other domains. Each study will be evaluated considering the ten items, and for each item the study will be classified as presenting low, unclear or high risk of bias. Publication bias will be accessed using funnel plots and the Egger´s regression test.

2.5. Collection of outcome data

Continuous outcome data from behavioral tests will be extracted by two independent authors (D.L. and A.P.), an any disagreement will be discussed with a third author (E.G.). Data will be extracted directly from the full-text article. When not reported in enough details, extraction will be done by graph estimation using a digital ruler, as previously described (23, 24). If both methods are not viable, the authors will be contacted. Two attempts will be performed within a two-week interval. If no response is obtained, the article will be excluded. Each included article will be divided in experiments, defined as any case when a control group is compared to an experimental group. If the same animals were subjected to more than one behavioral experiment from the same category (e.g. two distinct locomotion tests), both data will be extracted and the one with the largest effect size will be included in the analysis. In addition, only one outcome will be extracted from each behavioral experiment. Whenever the article reports multiple outcomes from the same behavioral test, the extraction will be performed according to a relevance rank organized by one reviewer. Variables from each behavioral test will be ranked subjectively according to their importance, and the one ranked highest will be extracted. If the same animals were evaluated more than once in the same behavioral test, the last one will be selected for data extraction. If the manuscript divides the results by time, the first time point will be selected for extraction.

2.6. Data analysis

2.6.1. Meta-analysis

Studies will be grouped according to the behavioral outcomes reported (locomotion, attention, impulsivity or memory), and a meta-analysis will be performed for each group. A minimum of three experiments will be required in order to conduct the meta-analysis. Pooled effect sizes will be determined with standardized mean differences using Hedge´s G method

with random-effects, allowing the comparison among distinct behavioral tests. The significance of pooled effect sizes will be determined using the Z-test. Since four statistical hypothesis tests will be performed, a Bonferroni correction will be applied in order to control the family-wise type I error rate, and a p-value ≤ 0.0125 will be considered as statistically significant. Individual study weights will be obtained using the inverse of the variance. Data will be transformed in order to obtain positive values for decreased impulsive behavior and for increased attentional or memory performances.

Heterogeneity between studies will be assessed using both the Chi^2 and the I^2 tests, and a p-value ≤ 0.1 will be considered as statistically significant. A I^2 value on the order of 25%, 50% and 75% will be considered as low, moderate, and high heterogeneity, respectively (25). Standardized mean differences and heterogeneity values will be obtained using Review Manager (RevMan) version 5.3 (Copenhagen: The Nordic Cochrane Centre, The Cochrane Collaboration, 2014).

2.6.2. Meta-regression

A meta-regression will be conducted for each behavioral outcome in order to evaluate potential sources of variability among studies. The following covariates will be selected based on biological plausibility and added to a univariate random-effects meta-regression model: age of animals; route of drug administration; MPH total dosage. Age of animals will be categorized in "adolescent" or "adult" before being included in the model, since some studies may not report the age in days or weeks. Animals 60 days old or more will be defined as "adults", while animals 28 to 60 days old will be defined as "adolescents" (26). Route of drug administration will be added as a categorical variable using dummy variables. MPH total dosage will be calculated by multiplying the dosage received in mg/kg by the number of administrations per day and by the total days of treatment and added as a continuous variable.

Covariates associated with the outcome with a $p \leq 0.1$ in a univariate analysis will be included in a final multivariate meta-regression model. Since up to four statistical hypothesis tests will be performed in the multivariate model, a Bonferroni correction will be applied in order to control the family-wise type I error rate. Studies with missing values will be excluded from the meta-regression analysis. Meta-regression will be conducted using Stata 13.0 (College Station, TX: StataCorp LP), as previously described (27).

2.6.3. Sensitivity analysis

Sensitivity analyses will be performed in order to evaluate effect size differences related to main methodological decisions in our manuscript. The following sensitivity analyses will be performed for each behavioral category: (1) the jackknife method, a common procedure used to test the stability of the outcome after excluding one result at a time (28); (2) including only effect sizes extracted from the same behavioral test, with a minimum of three experiments; (3) including studies with crossover designs; (4) including only one MPH dosage at a time, with a minimum of three studies using the same dosage; (5) excluding studies presenting a concerning risk of bias, defined as either a high risk of bias in one category or an unclear risk of bias in 7 categories or more.

The objectives of our sensitivity analyses will be to (1) observe if any study skews the overall result; (2) evaluate if the effects of MPH are different for distinct behavioral tests; (3) evaluate possible changes in effect sizes due to a carryover effect from crossover studies; (4) observe if the effects of MPH are different for distinct dosages; (5) evaluate the impact of studies with a high risk of bias. Sensitivity analyses will not be corrected for multiple comparison, and the results will be interpreted as exploratory. A p-value ≤ 0.05 will be considered statistically significant for these analyses.

3. Discussion

The pathophysiology of ADHD is still mostly unknown, and a valid animal model is believed to be essential in order to advance this knowledge. Although the SRH are the most widely used animal model of ADHD, its validity has been questioned by different authors. The poor methodological description, high heterogeneity, and high probability of bias observed in preclinical research seems to be a relevant factor for the interpretation of the published literature (29). In this sense, an overall estimate of the available data obtained using systematic reviews and meta-analyses has become more important over the years. Although they have been already used in the last decades in order to guide clinical practice, systematic reviews and meta-analyses are relatively new in preclinical research (30).

The predictive validity represents the ability of the animal model to respond to well documented treatments for the disorder, and is essential for the characterization of a valid animal model (11). In this sense, understanding the effects of MPH in the SHR is of great importance since MPH is the pharmacological treatment most commonly used in ADHD. However, we also want to highlight that other pharmacological approaches available for ADHD will not be included in our analysis, thus limiting our conclusions. In the SHR, distinct behavioral responses have been reported after a treatment with MPH. For instance, van den Bergh, Bloemarts (14) reported no effects of MPH in the animal model of ADHD, while Tamburella, Micale (31) reported increased locomotion after MPH administration. A deleterious effect of MPH in attentional performance of SHR has also been demonstrated (32). On the other hand, these results are contradicted by several studies showing decreased locomotion (33-36) and increased attention (33, 37-39) after treatment. Even though these divergences might be explained by methodological aspects including MPH dosage, route of drug administration and age of animals, no study has been conducted in order to evaluate this hypothesis.

By conducting this study, we expect to: (1) provide an overall estimate of the effect size of MPH on behavioral outcomes when administered to the SHR; (2) provide an estimate of the heterogeneity present in the published literature; and (3) identify covariates potentially related to this heterogeneity using meta-regression and sensitivity analyses. Our main hypothesis are: (1) MPH treatment will decrease locomotion and impulsivity, and increase attention and memory performance of the SHR; (2) a high heterogeneity will be found; (3) age of animals, route of drug administration and MPH total dosage are going to be statistically related to the effects.

A detailed description of the methodology to be applied in systematic reviews and meta-analyses has many advantages, including expert external opinion on the methods, prevention of data fishing, and the possibility of helping researchers in future applications. Additionally, protocol publication is an opportunity to discuss potential sources of bias that may be present in the future. In this sense, the following factors should be highlighted from our methodology. First, we are going to include only one data point for each behavioral test, which will be selected based on a subjective relevance rank. Although distinct approaches were available for the extraction (e.g. the combination of multiple variables into a single data point), we believe that the use of only one variable may ensure increased consistency among results. It is known that the same behavioral test can be performed in order to investigate distinct behavioral parameters. As an example, the open field test can be used to measure locomotion (40), anxiety-related behavior (41), or spatial memory (42), based primarily on which variable is collected. Therefore, even though our approach may present a risk of bias for being based on a subjective evaluation of importance, we believe that the grouping of multiple variables into a single data point would be an even greater threat since it would combine distinct information, thus increasing heterogeneity. Another important aspect is

related to our meta-regression model. As previously mentioned, studies with missing values will be excluded from the analysis, which can bias the final results.

To sum up, we believe that the results obtained from this study will be valuable in order to corroborate the predictive validity of SHR as an animal model of ADHD. Additionally, our results will provide data on the methodological strengths of published literature, which may aid the development of new experimental designs. In this way, our study will provide a background for the development of new research and for the advance of knowledge in the area.

4. References

1. Faraone SV, Asherson P, Banaschewski T, Biederman J, Buitelaar JK, Ramos-Quiroga JA, et al. Attention-deficit/hyperactivity disorder. Nature reviews Disease primers. 2015;1:15020.
2. Danckaerts M, Sonuga-Barke EJ, Banaschewski T, Buitelaar J, Dopfner M, Hollis C, et al. The quality of life of children with attention deficit/hyperactivity disorder: a systematic review. European child & adolescent psychiatry. 2010;19(2):83-105.
3. Man KKC, Coghill D, Chan EW, Lau WCY, Hollis C, Liddle E, et al. Association of Risk of Suicide Attempts With Methylphenidate Treatment. JAMA psychiatry. 2017;74(10):1048-55.
4. Biederman J, Faraone SV. The effects of attention-deficit/hyperactivity disorder on employment and household income. MedGenMed : Medscape general medicine. 2006;8(3):12.
5. Dalsgaard S, Ostergaard SD, Leckman JF, Mortensen PB, Pedersen MG. Mortality in children, adolescents, and adults with attention deficit hyperactivity disorder: a nationwide cohort study. Lancet (London, England). 2015;385(9983):2190-6.
6. Geschwind DH, Flint J. Genetics and genomics of psychiatric disease. Science (New York, NY). 2015;349(6255):1489-94.
7. Demontis D, Walters RK, Martin J, Mattheisen M, Als TD, Agerbo E, et al. Discovery Of The First Genome-Wide Significant Risk Loci For ADHD. bioRxiv. 2017.
8. Hoogman M, Bralten J, Hibar DP, Mennes M, Zwiers MP, Schweren LSJ, et al. Subcortical brain volume differences in participants with attention deficit hyperactivity disorder in children and adults: a cross-sectional mega-analysis. The lancet Psychiatry. 2017;4(4):310-9.
9. Hart H, Radua J, Nakao T, Mataix-Cols D, Rubia K. Meta-analysis of functional magnetic resonance imaging studies of inhibition and attention in attention-deficit/hyperactivity disorder: exploring task-specific, stimulant medication, and age effects. JAMA psychiatry. 2013;70(2):185-98.
10. Nestler EJ, Hyman SE. Animal models of neuropsychiatric disorders. Nature neuroscience. 2010;13(10):1161-9.
11. van der Staay FJ, Arndt SS, Nordquist RE. Evaluation of animal models of neurobehavioral disorders. Behavioral and brain functions : BBF. 2009;5:11.
12. Sagvolden T, Russell VA, Aase H, Johansen EB, Farshbaf M. Rodent models of attention-deficit/hyperactivity disorder. Biological psychiatry. 2005;57(11):1239-47.
13. Russell VA. Overview of animal models of attention deficit hyperactivity disorder (ADHD). Current protocols in neuroscience. 2011;Chapter 9:Unit9.35.
14. van den Bergh FS, Bloemarts E, Chan JS, Groenink L, Olivier B, Oosting RS. Spontaneously hypertensive rats do not predict symptoms of attention-deficit hyperactivity disorder. Pharmacology, biochemistry, and behavior. 2006;83(3):380-90.
15. Wickens JR, Hyland BI, Tripp G. Animal models to guide clinical drug development in ADHD: lost in translation? British journal of pharmacology. 2011;164(4):1107-28.
16. Faraone SV, Biederman J, Spencer TJ, Aleardi M. Comparing the efficacy of medications for ADHD using meta-analysis. MedGenMed : Medscape general medicine. 2006;8(4):4.
17. Shier AC, Reichenbacher T, Ghuman HS, Ghuman JK. Pharmacological treatment of attention deficit hyperactivity disorder in children and adolescents: clinical strategies. Journal of central nervous system disease. 2013;5:1-17.

18. Wilens TE, Morrison NR, Prince J. An update on the pharmacotherapy of attention-deficit/hyperactivity disorder in adults. Expert Rev Neurother. 2011;11(10):1443-65.
19. Pliszka SR. Pharmacologic treatment of attention-deficit/hyperactivity disorder: efficacy, safety and mechanisms of action. Neuropsychology review. 2007;17(1):61-72.
20. de Vries RBM, Hooijmans CR, Langendam MW, van Luijk J, Leenaars M, Ritskes-Hoitinga M, et al. A protocol format for the preparation, registration and publication of systematic reviews of animal intervention studies. Evidence-based Preclinical Medicine. 2015;2(1):1-9.
21. Curtin F, Elbourne D, Altman DG. Meta-analysis combining parallel and cross-over clinical trials. III: The issue of carry-over. Statistics in medicine. 2002;21(15):2161-73.
22. Hooijmans CR, Rovers MM, de Vries RB, Leenaars M, Ritskes-Hoitinga M, Langendam MW. SYRCLE's risk of bias tool for animal studies. BMC medical research methodology. 2014;14:43.
23. Pires GN, Bezerra AG, Tufik S, Andersen ML. Effects of experimental sleep deprivation on anxiety-like behavior in animal research: Systematic review and meta-analysis. Neuroscience and biobehavioral reviews. 2016;68:575-89.
24. Pires GN, Bezerra AG, Tufik S, Andersen ML. Effects of acute sleep deprivation on state anxiety levels: a systematic review and meta-analysis. Sleep medicine. 2016;24:109-18.
25. Higgins JP, Thompson SG. Quantifying heterogeneity in a meta-analysis. Statistics in medicine. 2002;21(11):1539-58.
26. Spear LP. The adolescent brain and age-related behavioral manifestations. Neuroscience and biobehavioral reviews. 2000;24(4):417-63.
27. Harbord RM, Higgins JPT. Meta-regression in Stata. Stata Journal. 2008;8(4):493-519.
28. Miller RG. The jackknife-a review. Biometrika. 1974;61(1):1-15.
29. Kilkenny C, Parsons N, Kadyszewski E, Festing MF, Cuthill IC, Fry D, et al. Survey of the quality of experimental design, statistical analysis and reporting of research using animals. PloS one. 2009;4(11):e7824.
30. Sena ES, Currie GL, McCann SK, Macleod MR, Howells DW. Systematic reviews and meta-analysis of preclinical studies: why perform them and how to appraise them critically. Journal of cerebral blood flow and metabolism : official journal of the International Society of Cerebral Blood Flow and Metabolism. 2014;34(5):737-42.
31. Tamburella A, Micale V, Mazzola C, Salomone S, Drago F. The selective norepinephrine reuptake inhibitor atomoxetine counteracts behavioral impairments in trimethyltin-intoxicated rats. European journal of pharmacology. 2012;683(1-3):148-54.
32. Aspide R, Fresiello A, de Filippis G, Gironi Carnevale UA, Sadile AG. Non-selective attention in a rat model of hyperactivity and attention deficit: subchronic methylphenydate and nitric oxide synthesis inhibitor treatment. Neuroscience and biobehavioral reviews. 2000;24(1):59-71.
33. Hong Q, Zhang M, Pan XQ, Guo M, Li F, Tong ML, et al. Prefrontal cortex Homer expression in an animal model of attention-deficit/hyperactivity disorder. Journal of the neurological sciences. 2009;287(1-2):205-11.
34. Somkuwar SS, Kantak KM, Bardo MT, Dwoskin LP. Adolescent methylphenidate treatment differentially alters adult impulsivity and hyperactivity in the Spontaneously Hypertensive Rat model of ADHD. Pharmacology, biochemistry, and behavior. 2016;141:66-77.

35. Kim H, Heo HI, Kim DH, Ko IG, Lee SS, Kim SE, et al. Treadmill exercise and methylphenidate ameliorate symptoms of attention deficit/hyperactivity disorder through enhancing dopamine synthesis and brain-derived neurotrophic factor expression in spontaneous hypertensive rats. Neuroscience letters. 2011;504(1):35-9.
36. Yang MT, Lu DH, Chen JC, Fu WM. Inhibition of hyperactivity and impulsivity by carbonic anhydrase inhibitors in spontaneously hypertensive rats, an animal model of ADHD. Psychopharmacology. 2015;232(20):3763-72.
37. Cheng JT, Li JS. Intra-orbitofrontal cortex injection of haloperidol removes the beneficial effect of methylphenidate on reversal learning of spontaneously hypertensive rats in an attentional set-shifting task. Behavioural brain research. 2013;239:148-54.
38. Fox GB, Pan JB, Esbenshade TA, Bennani YL, Black LA, Faghih R, et al. Effects of histamine H(3) receptor ligands GT-2331 and ciproxifan in a repeated acquisition avoidance response in the spontaneously hypertensive rat pup. Behavioural brain research. 2002;131(1-2):151-61.
39. Robinson AM, Eggleston RL, Bucci DJ. Physical exercise and catecholamine reuptake inhibitors affect orienting behavior and social interaction in a rat model of attention-deficit/hyperactivity disorder. Behavioral neuroscience. 2012;126(6):762-71.
40. Tatem KS, Quinn JL, Phadke A, Yu Q, Gordish-Dressman H, Nagaraju K. Behavioral and Locomotor Measurements Using an Open Field Activity Monitoring System for Skeletal Muscle Diseases. Journal of Visualized Experiments : JoVE. 2014(91).
41. Lecorps B, Rodel HG, Feron C. Assessment of anxiety in open field and elevated plus maze using infrared thermography. Physiology & behavior. 2016;157:209-16.
42. Dvorkin A, Benjamini Y, Golani I. Mouse Cognition-Related Behavior in the Open-Field: Emergence of Places of Attraction. PLOS Computational Biology. 2008;4(2):e1000027.
